# Prone positioning in acute respiratory distress syndrome during venovenous extracorporeal membrane oxygenation

**DOI:** 10.1186/s13054-021-03760-w

**Published:** 2021-10-18

**Authors:** Shiping Zhu

**Affiliations:** Department of Respiratory Medicine, Hangzhou Hospital of Traditional Chinese Medicine, No. 453, Tiyuchang Road, Hangzhou, 310000 Zhejiang China

**Keywords:** ARDS, Prone positioning, Mortality, Meta-analysis

**Dear Editor,**

We read with respect the recent study by Dr. Poon [1], which investigated the potential benefit of prone positioning (PP) during venovenous extracorporeal membrane oxygenation (ECMO) in patients with acute respiratory distress syndrome (ARDS). Total of 11 studies were included and the pooled result showed a non-significant increasing trend of survival rate in patients receiving PP during ECMO (RR 1.2, 95% CI 0.9–1.5). We would like to add some comments.

First, in the forest plot, we noted that the result from Garcia-2020’s study was significantly different from others. We performed a sensitivity analysis by excluding Garcia-2020’s study (Fig. 1), and the pooled result became statistically significant (RR 1.28, 95% CI 1.08–1.52). We believe several reasons may help to explain this finding. 1 > In Garcia-2020’s study, the overall mortality rate was significantly higher than others (85% vs. 30–60%), which suggested potential heterogeneity within these ARDS cohorts. Therefore, whether PP during ECMO presented different efficacy in different ARDS phenotypes needs to be further investigated. 2 > PP during ECMO is still not routinely applied to patients during ECMO, due to risk of life-threatening complications, such as cannula dislodgement. In all these included studies, the indications for PP differed significantly. In Garcia-2020’s study, PP was only used in case of severe hypoxemia or extensive lung consolidation, which generated an inter-relationship between PP and disease severity due to selection bias. However, in Giani-2020’s and Schmidt’s studies, PP is routinely performed or encouraged during ECMO. The indications in Chaplin-2020, Guervilly-2020, Yang-2021 and Rilinge-2020’s studies were unclear. Therefore, we suggest that these conditions should be considered when interpreting the pooled result of the current study.

**Fig. 1:**
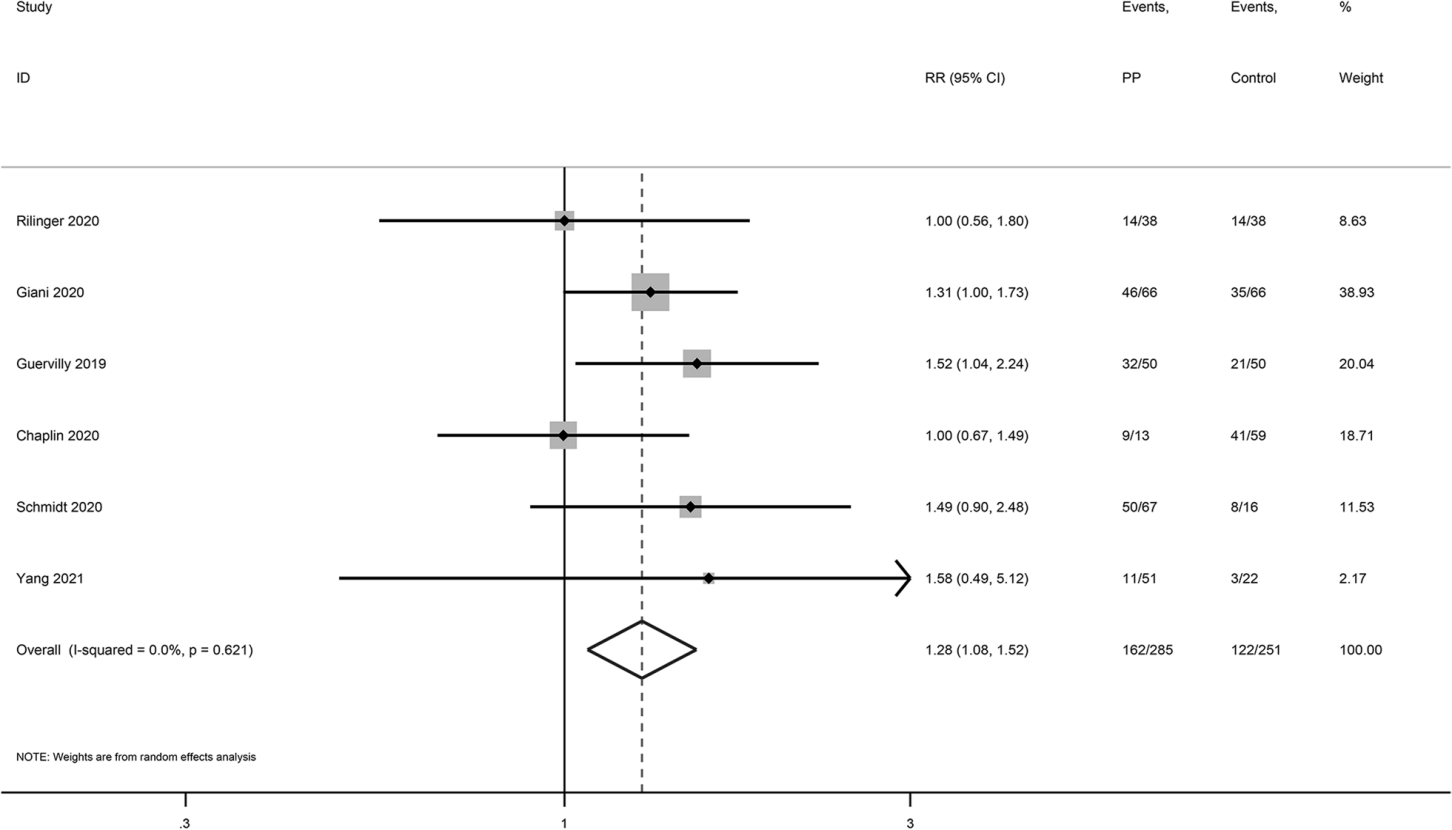
Forest plot showing the efficacy of PP during ECMO in patients with ARDS. Note: Garcia-2020’s study was excluded from this pooled result. Note: ARDS acute respiratory distress syndrome; ECMO venovenous extracorporeal membrane oxygenation; PP prone positioning

Second, a meta-analysis aims to pool studies with similar design, cohort, intervention, and outcomes. This also one reason for the debate that whether observational studies and randomized controlled studies should be included in one meta-analysis [2, 3]. In the current study, both the unadjusted findings from four studies and results after propensity score matching (PSM) from three studies were included in one forest plot. We suggest that the unadjusted findings and adjusted result (PSM or regression) should be separated [4].

## Data Availability

Not applicable.
