# Supplementary material for: Prone positioning in acute respiratory distress syndrome during venovenous extracorporeal membrane oxygenation
Source: Crit Care. 2021 Oct 18;25:361. doi: 10.1186/s13054-021-03760-w (PMC8522121; doi:10.1186/s13054-021-03760-w)
Supplement: Related texts

# Prone Positioning During Venovenous Extracorporeal Membrane Oxygenation for Acute Respiratory Distress Syndrome: Reply

**Authors:** Wynne Hsing Poon, Kollengode Ramanathan, Ryan Ruiyang Ling, Matthieu Schmidt, Kiran Shekar

**Affiliations:** 2 grid.4280.e 0000 0001 2180 6431 Yong Loo Lin School of Medicine, National University of Singapore, Singapore, Singapore; 3 grid.412106.0 0000 0004 0621 9599 Cardiothoracic Intensive Care Unit, National University Heart Centre, National University Hospital, Singapore, Singapore; 4 grid.411439.a 0000 0001 2150 9058 Service de Médecine Intensive-Réanimation, Institut de Cardiologie, Assistance Publique-Hôpitaux de Paris, Hôpital Pitié-Salpêtrière, Paris, France; 5 grid.411439.a 0000 0001 2150 9058 Sorbonne Université, GRC 30, Reanimation Et Soins Intensifs du Patient en Insuffisance Respiratoire aigüE, AP-HP, Hôpital de La Pitié Salpêtrière, 75013 Paris, France; 6 grid.415184.d 0000 0004 0614 0266 Adult Intensive Care Services, Prince Charles Hospital, Brisbane, QLD Australia; 7 grid.1024.7 0000000089150953 Queensland University of Technology, Brisbane, Australia; 8 grid.1003.2 0000 0000 9320 7537 University of Queensland, Brisbane, Australia; 9 grid.1033.1 0000 0004 0405 3820 Bond University, Gold Coast, QLD Australia

Wynne Hsing Poon and Kollengode Ramanathan have contributed equally to this work

We thank Dr Zhu for the attention and valuable comments provided regarding our article. The indications for prone positioning (PP) during extracorporeal membrane oxygenation (ECMO) were indeed variable across studies, as outlined in our Supplementary Table 2 of our original manuscript [1]. Based on our a priori criteria for sensitivity analyses (Joanna Briggs Institute score < 8), the exclusion of study by Garcia et al., which primarily focused on COVID-19 patients, was not indicated. While we note that the sensitivity analysis suggested found significant survival benefits, this would be an unplanned post-hoc analysis, which should be considered exploratory and interpreted carefully [5].

Despite varied indications for PP, the baseline PF ratio reported by Garcia et al. (82.3 ± 22.5) was relatively similar to Giani et al. (73 ± 29) and Schmidt et al. (all patients, 60 [54–68]), suggesting a similar degree of refractory hypoxemia despite ECMO support. Additionally, metaregression analysis found that PF ratio did not independently influence patient survival. Though establishing an inter-relationship between disease severity or ARDS phenotypes and concurrent PP during ECMO goes beyond the scope of our meta-analysis, we wholly agree with Dr. Zhu that further studies should be conducted to shed light on these thought-provoking insights.

A plausible reason for the increased mortality reported by Garcia et al. could be related to the planning and provision of ECMO services during the COVID-19 pandemic [6]. With a pandemic-stricken and overwhelmed healthcare system, it is possible that patient outcomes are affected. Our subgroup analysis, albeit insignificant, also found a trend towards decreased survival in patients with COVID-19 (37%) compared to those without (64%).

This meta-analysis of observational studies aimed to summarize all available information on the application of PP with ECMO. The Cochrane recommendations suggest that in a meta-analysis of non-randomized data, adjusted results should be collected where possible [7]. However, we appreciate the suggestion to separate adjusted and unadjusted data, for which an additional analysis found no significant difference between groups (Table 1).

**Table 1:** Subgroup analysis based on study data type for chance of cumulative survival

Data type	No. of studies	RR (95% confidence interval)	*P* value	*P* value for interaction
Non-PSM	4	0.98 [0.54–1.79]	0.95	0.354
PSM	3	1.33 [1.07–1.64]	0.0086	

*PSM* Propensity score matched

While the adjusted data suggest significant survival benefit, this should be interpreted with caution as the conclusions are based on observational study-level data from three studies. As we understand ARDS and its therapeutic interventions better, the clinical outcomes of patients are likely to improve. Clinical decisions for these patients should evolve with time, while being evidence based. Better evidence should be obtained from well-conducted clinical trials to elucidate whether PP during ECMO demonstrates a survival benefit and identify patients who are most likely to benefit.
